# Assembling the anaerobic gamma-butyrobetaine to TMA metabolic pathway in *Escherichia fergusonii* and confirming its role in TMA production from dietary *L-*carnitine in murine models

**DOI:** 10.1128/mbio.00937-23

**Published:** 2023-09-22

**Authors:** Mohammed Dwidar, Jennifer A. Buffa, Zeneng Wang, Akeem Santos, Aaron N. Tittle, Xiaoming Fu, Adeline M. Hajjar, Joseph A. DiDonato, Stanley L. Hazen

**Affiliations:** 1 Department of Cardiovascular and Metabolic Sciences, Lerner Research Institute, Cleveland Clinic, Cleveland, Ohio, USA; 2 Center for Microbiome and Human Health, Cleveland Clinic, Cleveland, Ohio, USA; 3 Cleveland Clinic Lerner College of Medicine, Case Western Reserve University, Cleveland, Ohio, USA; 4 Department of Cardiovascular Medicine, Heart, Vascular, and Thoracic Institute, Cleveland Clinic, Cleveland, Ohio, USA; Rutgers, The State University of New Jersey, New Brunswick, New Jersey, USA; Harvard University, Cambridge, Massachusetts, USA

**Keywords:** TMA, TMAO, *L-carnitine*, Emergencia timonensis, atherosclerosis, cardiovascular disease

## Abstract

Trimethylamine-N-oxide (TMAO) is a major pro-atherogenic and pro-thrombotic metaorganismal molecule produced through the initial conversion of the dietary *L*-carnitine and other precursors into trimethylamine (TMA). We recently identified a dual-microbe anaerobic pathway for the metabolism of *L-*carnitine into TMA, in which the widely distributed *cai* operon in *Enterobacteriaceae* converts *L-*carnitine into gamma-butyrobetaine (γBB), followed by the degradation of γBB into TMA by the relatively rare gamma-butyrobetaine utilization (*gbu*) gene cluster present in *Emergencia timonensis* and few other related microbes. Studies of this pathway in animal models, however, have been limited by the lack of single microbes harboring the whole *L-*carnitine→γBB→TMA transformation pathway. Such recombinant microbes would both serve as a tool to further prove the contribution of this pathway to gut microbial TMA production and for future *in vivo* studies investigating the diet linkage to cardiovascular disease and the involvement of the TMAO pathway in this linkage. Here, we recapitulate the whole pathway in a single microbe by cloning the *E. timonensis gbu* gene cluster into *Escherichia fergusonii*, which naturally harbors the *cai* operon. We then show that the native *E. timonensis* GroES/GroEL-like proteins are needed for the proper functioning of the *gbu* cluster at 37°C. Finally, we demonstrate that inoculating germ-free mice with this recombinant *E. fergusonii* strain is sufficient to raise serum TMAO to pathophysiological levels upon dietary *L-*carnitine supplementation. The recombinant *E. fergusonii* strain developed will be a useful tool to facilitate future studies on the role of anaerobic gut microbial *L-*carnitine metabolism in cardiovascular and metabolic diseases.

**IMPORTANCE:**

The key atherosclerotic TMAO originates from the initial gut microbial conversion of *L*-carnitine and other dietary compounds into TMA. Developing therapeutic strategies to block gut microbial TMA production needs a detailed understanding of the different production mechanisms and their relative contributions. Recently, we identified a two-step anaerobic pathway for TMA production from *L*-carnitine through initial conversion by some microbes into the intermediate γBB which is then metabolized by other microbes into TMA. Investigational studies of this pathway, however, are limited by the lack of single microbes harboring the whole pathway. Here, we engineered *E. fergusonii* strain to harbor the whole two-step pathway and optimized the expression through cloning a specific chaperone from the original host. Inoculating germ-free mice with this recombinant *E. fergusonii* is enough to raise serum TMAO to pathophysiological levels upon L-carnitine feeding. This engineered microbe will facilitate future studies investigating the contribution of this pathway to cardiovascular disease.

## OBSERVATION

Trimethylamine-N-oxide (TMAO) is a key pro-atherogenic and pro-thrombotic molecule produced through a metaorganismal pathway, in which the gut microbes first degrade trimethylamine (TMA) containing nutrients, including *L-*carnitine and phosphatidylcholine, into TMA (1 – 3). TMA is then converted by host flavin-containing monooxygenases into TMAO (4). Previously, our group proposed a novel, anaerobic multi-step gut microbial pathway for TMA production from dietary *L-*carnitine (3, 5 – 7). In this pathway, dietary *L-*carnitine is first converted into gamma-butyrobetaine (γBB) by microbes possessing the *cai* operon, which is widely distributed among members of the family *Enterobacteriaceae*, including *Escherichia*, *Citrobacter*, *Proteus*, and others (8, 9). The intermediate γBB is then converted into TMA by another group of microbes that include *Emergencia timonensis* and a handful of closely related species belonging to the Clostridia class (6, 7).

In recent studies, we reported that the novel *gbu* (gamma-butyrobetaine utilization) gene cluster in *E. timonensis* plays a critical role in the conversion of γBB to TMA and the abundance of *gbu* is increased in the setting of a diet rich in red meat as a protein source (7). In parallel, studies from another group also identified the *gbu* gene cluster as contributing to the gut microbial transformation of *L-*carnitine-derived γBB into TMA (10). Human dietary intervention experiments have shown that the fecal abundance of the *gbuA* gene (the first gene in the *gbu* gene cluster) not only is enhanced with increased dietary red meat but also is reduced following conversion to either a white meat or a non-meat isocaloric diet (7). Furthermore, it was previously shown that the red meat diet increases TMA and TMAO production from *L*-carnitine but not choline (11). A red meat-rich diet is a known contributor to cardiovascular disease risks (12 – 14) and has been similarly linked to risks for alternative diseases including colorectal cancer (15) and other cancer types (16). The ability to recapitulate the multi-step multi-microbial *L-*carnitine → γBB → TMA transformation in murine models using a single microbe possessing both *cai* and *gbu* gene clusters would therefore serve as a tool to further investigate the role of dietary red meat and the TMAO pathway in animal models of disease (17). Thus, in the present study, we aimed to investigate whether combining the *cai* and *gbu* gene clusters into a single genetically tractable microbe will allow this engineered microbe to convert dietary *L*-carnitine into TMA (and eventual TMAO generation) in murine models.

Given the poor colonization of *E. timonensis* in the murine host we observed in our previous study (7), and the current unavailability of the needed genetic tools to manipulate *E. timonensis* or the other related microbes harboring the *gbu* cluster, we opted to clone the *gbu* gene cluster, instead, into one of the microbes capable of performing the first step (*L-*carnitine → γBB). In previous studies employing combinatorial cloning and functional analyses (7), we showed that when the four *gbu* genes (*gbuA*, *B*, *C*, and *E*) were cloned from *E. timonensis* into a pET vector and transformed in *Escherichia coli* BL21/DE3 strain, TMA production from γBB occurred only at temperatures of 18°C or below, while no production was observed at 37°C in recombinant *E. coli*. Given that within the human gut, *E. timonensis* efficiently converts ɣBB into TMA at body temperature (37°C), this indicates that one or more additional proteins (presumably a chaperone) are needed for the proper folding or stability of the gbu proteins at 37°C. Identifying and expressing this additional chaperone, therefore, is needed to enable investigational studies of this metaorganismal pathway in a recombinant microbe within a murine host.

**Fig 1:**
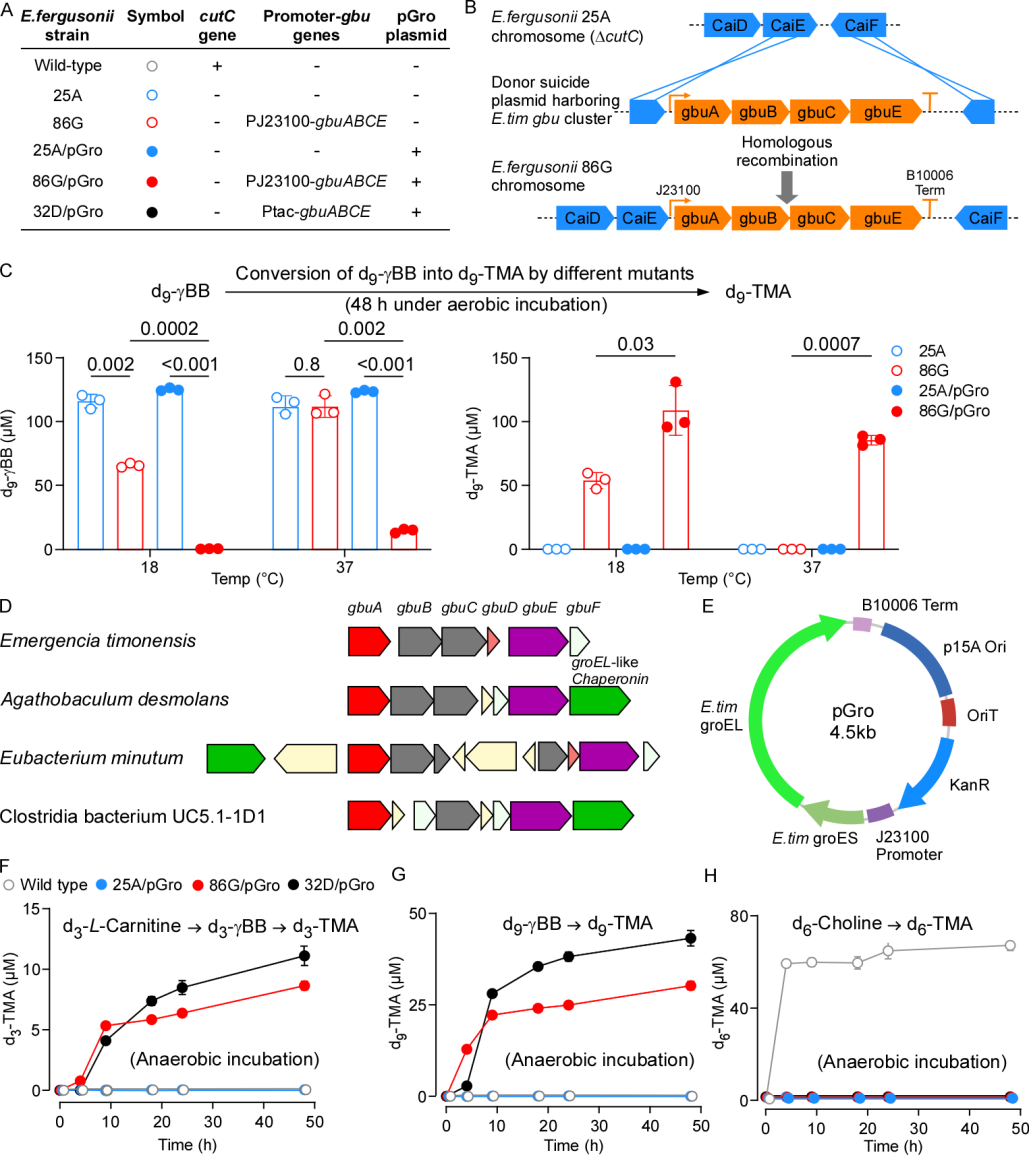
Cloning and expression of the *gbu* gene cluster and *groES/groEL*–like chaperone genes from *E. timonensis* into *E. fergusonii*. (**A**) Scheme of the strains constructed in this study with their genotypes. (**B**) The *gbu* genes were cloned downstream of the *caiE* gene in *E. fergusonii* chromosome. (**C**) Harboring pGro plasmid enabled *E. fergusonii* 86G strain (∆*cutC*, PJ23100*-gbuABCE*) but not the control *E. fergusonii* 25A strain (∆*cutC*) to convert γBB to TMA at 37^ᴏ^C aerobically (*n* = 3). Reported are *P*-values from Welch *t*-test. (**D**) Gene neighborhood analysis for the *gbu* gene cluster showing the presence of a nearby chaperone *groEL*-like gene in *A. desmolans*, *E. minutum*, and *Clostridia* sp. UC5.1–1D1, but not in *E. timonensis*. (**E**) Map of the pGro plasmid. (F, G, and H) Time-wise TMA production from different substrates by *E. fergusonii* wild-type and mutant strains under anaerobic conditions. All strains were cultured anaerobically at 37°C in Luria-Bertani (LB) media supplemented with 120 µM of each of d_3_-*L*-carnitine, d_9_- γBB, and d_6_-choline. Samples were taken at time intervals for LC-MS/MS analyses (*n* = 5). Bars represent Mean ± SE.

In the current study, to address this unmet need, we first generated a knock-in mutant of *Escherichia fergusonii* ATCC 35469 (*E. fergusonii* mutant 86G) in which a synthetic operon containing *gbuA*, *B*, *C*, and *E* was inserted downstream of the native *CaiE* gene (Fig. 1A and B). This strain was chosen because it lacks the aerobic CntA/B pathway, which can convert the *L-*carnitine directly into TMA (18). *E. fergusonii*, however, harbors choline TMA lyase enzyme (CutC/D), which converts choline into TMA (19). Therefore, to ensure that the mutant *E. fergusonii* is unable to utilize other pathways for TMA production, the gain-of-function mutation was generated in a ∆*cutC E. fergusonii* mutant background (*E. fergusonii* mutant 25A). The synthetic operon expression was derived by the synthetic promoter J23100 (BBa_J23100, Biobricks). Incubating this mutant with d_9_-γBB under aerobic conditions confirmed that the four *gbu* genes were sufficient to produce d_9_-TMA from d_9_-γBB at 18°C, while no TMA was produced at 37°C (Fig. 1C). These results further confirmed that a molecular chaperone is needed for proper folding of one or more of the gbu cluster proteins at 37°C. Transforming and expressing each of the five commercial chaperone plasmids pG-KJE8, pGro7, pKJE7, pGTf2, and pTf16 (20, 21), which harbor different combinations of the *E. coli* chaperone genes *dnaK*, *dnaJ*, *grpE*, *groES*, *groEL*, and *tig*, did not result in TMA production at 37°C (data not shown). Examining the *gbu* cluster neighborhood in *E. timonensis* SN18, we could not identify a nearby gene likely to serve this function, so we next explored the *gbu* gene cluster neighborhood in other bacterial strains capable of producing TMA from γBB, including *Agathobaculum desmolans*, *Eubacterium minutum*, and Clostridia bacterium UC5.1–1D1 (7). Interestingly, we found a predicted *groEL*-like chaperonin that is associated with the *gbu* cluster in those three strains (Fig. 1D).

Blasting this chaperonin protein (locus tag: T363DRAFT_02358) from *A. desmolans* against *E. timonensis* SN18 found it to be 57% identical and 76% similar to the *E. timonensis* GroEL-like protein (WP_067536048). Likewise, *E. timonensis* GroEL-like protein showed 73% identity and 84% similarity to the corresponding locus tag of this chaperonin protein (Ga0349467_1214) in *E. minutum. E. timonensis* GroEL-like protein also showed 58% identity and 77% similarity to the corresponding locus tag of this chaperonin protein (Ga0100572_11685) in Clostridia bacterium UC5.1–1D1. Therefore, we next cloned the *E. timonensis* operon containing the putative *groEL*-like gene together with the upstream *groES*-like gene in plasmid (pGro; Fig. 1E) and transformed pGro plasmid into both *E. fergusonii* 25A (*∆cutC*) and *E. fergusonii* 86G (*∆cutC*, *gbuA,B,C,E+*) to make *E. fergusonii* 25A/pGro and *E. fergusonii* 86G/pGro mutants. After incubating both transformed strains with d_9_-γBB, we found that *E. fergusonii* 86G/pGro strain was capable of performing the γBB → TMA transformation at 37°C, indicating that this chaperone complex from *E. timonensis* is sufficient for proper folding of the gbu cluster (gbuA, B, C, and E) proteins (Fig. 1C).

In *E. fergusonii* and other related organisms, the *cai* operon, responsible for converting *L-*carnitine into γBB, is induced under anaerobic conditions and in the presence of *L-*carnitine (9). As expected, when the culture media were supplemented with d_3_-*L-*carnitine and upon incubating under anaerobic conditions, the *E. fergusonii* 86G/pGro strain was capable of not only converting d_9_-γBB into d_9_-TMA but also converting the supplemental d_3_-*L-*carnitine into d_3_-TMA through the d_3_-γBB intermediate, while it is still impaired in converting d_6_-choline into d_6_-TMA (Fig. 1F through H; Fig. S1). As *E. fergusonii* is a facultative anaerobe, the growth and consequently TMA production from d_9_-γBB by *E. fergusonii* 86G/pGro strain, were reduced under anaerobic conditions (Fig. 1G) when compared to TMA produced by this strain under aerobic conditions (Fig. 1C). To further increase TMA production by *E. fergusonii*, the J23100 promoter was replaced with the strong tac promoter to create the mutant *E. fergusonii* 32D/pGro. As expected, this mutant produced more of the respective TMA isotopologues from both d_3_-carnitine (making d_3_-TMA; Fig. 1F) and d_9_-γBB (making d_9_-TMA; Fig. 1G), albeit at the expense of slower growth rate (Fig. S2).

**Fig 2:**
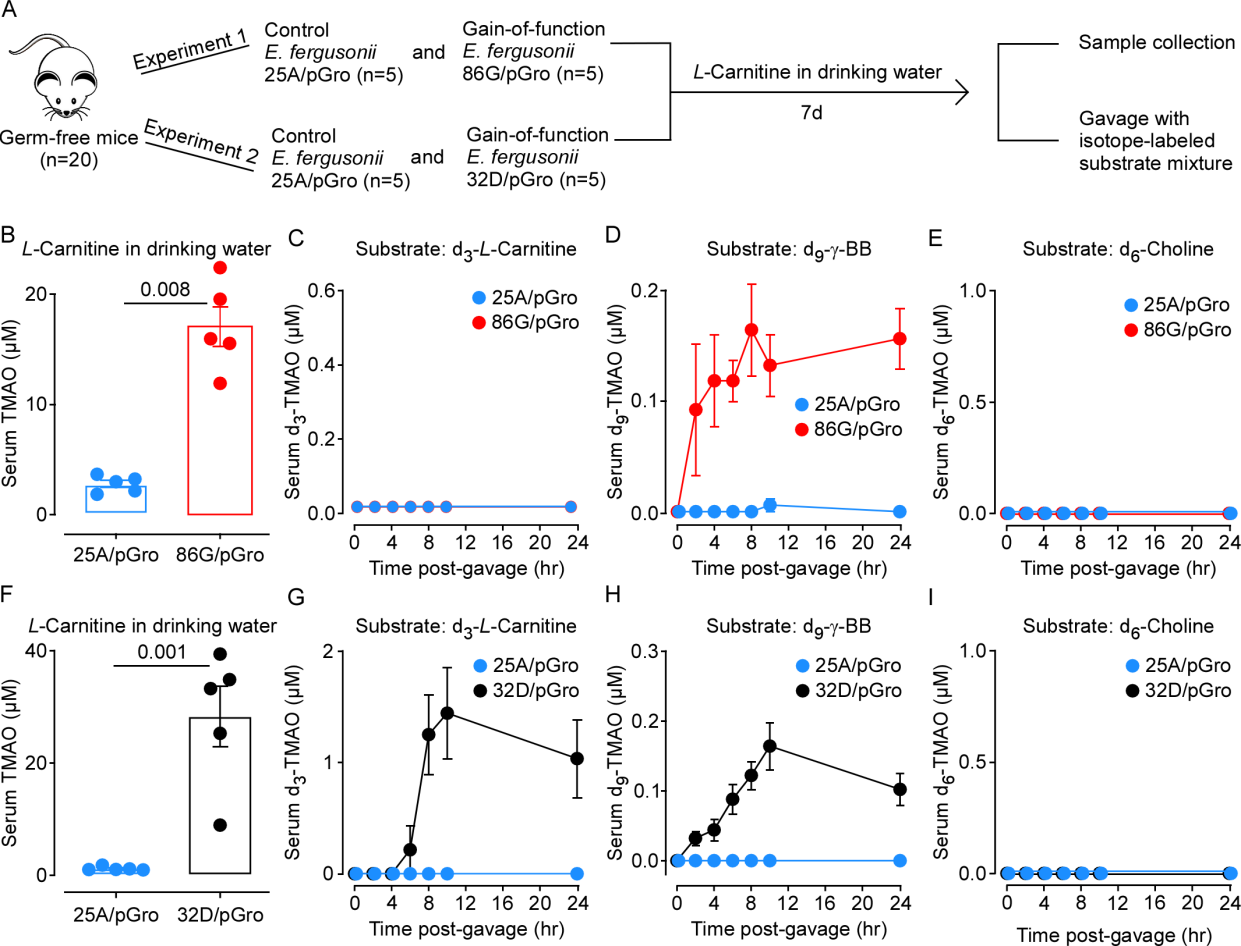
Colonization of germ-free mice with recombinant *E. fergusonii* expressing *gbuABCE* cassette and *groES/groEL*–like chaperone genes raises circulating TMAO levels following *L*-carnitine supplementation. **(A**) Schematic for the animal experiment flow. The gnotobiotic mice inoculated with control *E. fergusonii* 25A/pGro strain or the gain-of-function mutant (either *E. fergusonii* 86G/pGro or *E. fergusonii* 32D/pGro) were fed *L-c*arnitine in the drinking water (at 1.3%) for 1 week, and serum TMAO levels were measured. Both groups were then gavaged with a single bolus containing 22.4 micromoles each of d_6_-choline, d_3_-*L*-carnitine, and d_9_-γBB, and serum TMAO levels were measured at different time points after gavage. (**B–E**) The experiment was performed using gnotobiotic mice harboring the *E. fergusonii* 86G/pGro strain for the test group (*n* = 5) and the 25A/pGro strain for the control group (*n* = 5). (**F–I**) The same experiment was repeated using *E. fergusonii* 32D/pGro strain for the test group (*n* = 5) while using again *E. fergusonii* 25A/pGro for the control group (*n* = 5). Reported are *P*-values from the Mann Whitney test. Bars represent mean ± SE.

In additional studies, we tested the performance of these recombinant *E. fergusonii* mutants in murine models. Germ-free mice were first colonized with *E. fergusonii* 86G/pGro (which completes the *L*-carnitine to TMA transformation at 37°C) through oral gavage, while control mice were gavaged with the *E. fergusonii* 25A/pGro strain (which lacks the *gbu* gene cluster). The mice were then supplemented with either *L-*carnitine or γBB in the drinking water, and serum TMAO levels were measured following stabilization of the intestinal colonization 1 week later (Fig. 2A). We confirmed a similar degree of colonization between these two strains as determined by fecal bacterial load (Fig. S3A and B). On average, the 86G/pGro colonized mice supplemented with *L-*carnitine had TMAO levels around 17 µM (Fig. 2B), while those supplemented with γBB had an average TMAO level of only 1.8 µM (Fig. S4) indicating that direct feeding of *L*-carnitine but not γBB is effective in raising the serum TMAO level in 86G/pGro colonized mice. The mechanism(s) accounting for this difference are not clear but may reflect enhanced absorption (or metabolic transformation) of γBB in the proximal intestines, limiting the delivery of γBB to the more distal intestines.

To confirm that TMAO originates from the dietary *L-*carnitine → γBB → TMA pathway rather than other sources, *L*-carnitine administered mice were gavaged with a single bolus containing d_3_-*L-*carnitine, d_9_-γBB, and d_6_-choline, and the rate of substrate degradation (Fig. S5A through F) and accumulation of the corresponding TMAO isotopologues in the serum (Fig. 2C through E) and urine (Fig. S6A through C) was monitored over 24 h. As expected, no d_6_-TMA (from d_6_-choline) was detected in either the serum (Fig. 2E) or the urine (Fig. S6C) of either group, as the *cutC* gene is knocked out in both the control and the gain-of-function mutant. For the *E. fergusonii* 86G/pGro gain-of-function mutant, both d_3_-TMA and d_9_-TMA were detected in urine, albeit at low levels (Fig. S6A and B), while only d_9_-TMA but not d_3_-TMA was detected in serum (Fig. 2C and D). The absence of d_3_-TMA but not d_9_-TMA in the serum could be due to the competition of the gavaged d_3_-*L*-carnitine with the *L-*carnitine supplemented (1.3%) in the drinking water (for *L*-carnitine transporting and metabolizing enzymes in *E. fergusonii*).

In a subsequent experiment, mice were colonized with *E. fergusonii* 32D/pGro mutant (*∆cutC*, Ptac-*gbuA,B,C,E+*/pGro) vs the *E. fergusonii* 25A/pGro strain (control). Both strains again showed similar degrees of colonization after 1 week (Fig. S3C). As shown in Fig. 2F, TMAO levels were dramatically increased in the 32D/pGro colonized mice, reaching an average of around 28 µM in serum. Similarly, both groups of mice were gavaged with a single bolus of d_6_-choline, d_3_-carnitine, and d_9_-γBB to permit the simultaneous tracing of the rate of substrate degradation (Fig. S5G through L) and source of the different cognate TMA isotopologues. Both d_3_-TMAO and d_9_-TMAO (but not d_6_-TMAO) accumulated in the serum (Fig. 2G through I) and urine (Fig. S6D through F) of the 32D/pGro colonized mice, indicating that 32D/pGro strain is effective in producing TMA within the murine gut and is relatively superior to 86G/pGro strain in TMA production both *in vitro* and *in vivo* (Fig. 2B and F; Fig. S6).

In conclusion, we successfully developed a gain-of-function system in *E. fergusonii* to recapitulate the microbial anaerobic pathway for the conversion of *L-*carnitine into TMA. Genetic engineering of *E. fergusonii* to harbor both the *cai* and *gbu* gene clusters together with the required GroES/EL-like chaperone resulted in recombinant commensal mutants (86G/pGro and 32D/pGro) that could complete both the *L*-carnitine to γBB and γBB to TMA transformations at 37°C, allowing the recapitulation of the pathway in gnotobiotic mice. Finally, these engineered strains will enable us and others to move closer to the principles of Koch’s Postulates and should prove useful in studies aimed at exploring the impact of a red meat-rich diet on the *L*-carnitine→→TMA conversion in murine models of cardiometabolic diseases. Importantly, the recombinant *E. fergusonii* strains we engineered constitutively express all the necessary genes for the conversion of γBB into TMA, which eliminates the need for the induction of this pathway *in vivo* through exogenous chemical inducers.

## Data Availability

GraphPad prism nine was used to generate all the figures and for statistical analyses. All source data for figures included in the manuscript were deposited as GraphPad Prism files in Zenodo repository (https://zenodo.org/record/8189569).

## SUPPLEMENTAL MATERIAL

Supplemental Information mbio.00937-23-s0001.pdf Supplemental methods and Fig. S1–S6.

ASM does not own the copyrights to Supplemental Material that may be linked to, or accessed through, an article. The authors have granted ASM a non-exclusive, world-wide license to publish the Supplemental Material files. Please contact the corresponding author directly for reuse.

## References

[1] Wang Z , Klipfell E , Bennett BJ , Koeth R , Levison BS , Dugar B , Feldstein AE , Britt EB , Fu X , Chung Y-M , Wu Y , Schauer P , Smith JD , Allayee H , Tang WHW , DiDonato JA , Lusis AJ , Hazen SL . 2011. Gut flora metabolism of phosphatidylcholine promotes cardiovascular disease. Nature 472:57–63. doi:10.1038/nature09922 21475195 PMC3086762

[2] Tang WHW , Wang Z , Levison BS , Koeth RA , Britt EB , Fu X , Wu Y , Hazen SL . 2013. Intestinal microbial metabolism of phosphatidylcholine and cardiovascular risk. N Engl J Med 368:1575–1584. doi:10.1056/NEJMoa1109400 23614584 PMC3701945

[3] Koeth Robert A , Wang Z , Levison BS , Buffa JA , Org E , Sheehy BT , Britt EB , Fu X , Wu Y , Li L , Smith JD , DiDonato JA , Chen J , Li H , Wu GD , Lewis JD , Warrier M , Brown JM , Krauss RM , Tang WHW , Bushman FD , Lusis AJ , Hazen SL . 2013. Intestinal microbiota metabolism of L-carnitine, a nutrient in red meat, promotes atherosclerosis. Nat Med 19:576–585. doi:10.1038/nm.3145 23563705 PMC3650111

[4] Bennett BJ , de Aguiar Vallim TQ , Wang Z , Shih DM , Meng Y , Gregory J , Allayee H , Lee R , Graham M , Crooke R , Edwards PA , Hazen SL , Lusis AJ . 2013. Trimethylamine-N-oxide, a metabolite associated with atherosclerosis, exhibits complex genetic and dietary regulation. Cell Metab 17:49–60. doi:10.1016/j.cmet.2012.12.011 23312283 PMC3771112

[5] Koeth R.A , Levison BS , Culley MK , Buffa JA , Wang Z , Gregory JC , Org E , Wu Y , Li L , Smith JD , Tang WHW , DiDonato JA , Lusis AJ , Hazen SL . 2014. Gamma-butyrobetaine is a proatherogenic intermediate in gut microbial metabolism of L-carnitine to TMAO. Cell Metab 20:799–812. doi:10.1016/j.cmet.2014.10.006 25440057 PMC4255476

[6] Wang Z , Bergeron N , Levison BS , Li XS , Chiu S , Jia X , Koeth RA , Li L , Wu Y , Tang WHW , Krauss RM , Hazen SL . 2019. Impact of chronic dietary red meat, white meat, or non-meat protein on Trimethylamine N-oxide metabolism and renal excretion in healthy men and women. Eur Heart J 40:583–594. doi:10.1093/eurheartj/ehy799 30535398 PMC6374688

[7] Buffa JA , Romano KA , Copeland MF , Cody DB , Zhu W , Galvez R , Fu X , Ward K , Ferrell M , Dai HJ , Skye S , Hu P , Li L , Parlov M , McMillan A , Wei X , Nemet I , Koeth RA , Li XS , Wang Z , Sangwan N , Hajjar AM , Dwidar M , Weeks TL , Bergeron N , Krauss RM , Tang WHW , Rey FE , DiDonato JA , Gogonea V , Gerberick GF , Garcia-Garcia JC , Hazen SL . 2022. The microbial gbu gene cluster links cardiovascular disease risk associated with red meat consumption to microbiota L-carnitine catabolism. Nat Microbiol 7:73–86. doi:10.1038/s41564-021-01010-x 34949826 PMC8732312

[8] Elssner T , Preusser A , Wagner U , Kleber HP . 1999. Metabolism of L(-)-carnitine by enterobacteriaceae under aerobic conditions. FEMS Microbiol Lett 174:295–301. doi:10.1111/j.1574-6968.1999.tb13582.x 10339822

[9] Eichler K , Buchet A , Lemke R , Kleber HP , Mandrand-Berthelot MA . 1996. Identification and characterization of the caiF gene encoding a potential transcriptional activator of carnitine metabolism in Escherichia coli. J Bacteriol 178:1248–1257. doi:10.1128/jb.178.5.1248-1257.1996 8631699 PMC177796

[10] Rajakovich LJ , Fu B , Bollenbach M , Balskus EP . 2021. Elucidation of an anaerobic pathway for metabolism of L-carnitine-derived gamma-butyrobetaine to trimethylamine in human gut bacteria. Proc Natl Acad Sci U S A 118:e2101498118. doi:10.1073/pnas.2101498118 34362844 PMC8364193

[11] Wang Z , Bergeron N , Levison BS , Li XS , Chiu S , Jia X , Koeth RA , Li L , Wu Y , Tang WHW , Krauss RM , Hazen SL . 2019. Impact of chronic dietary red meat, white meat, or non-meat protein on trimethylamine N-oxide metabolism and renal excretion in healthy men and women. Eur Heart J 40:583–594. doi:10.1093/eurheartj/ehy799 30535398 PMC6374688

[12] Pan A , Sun Q , Bernstein AM , Schulze MB , Manson JE , Stampfer MJ , Willett WC , Hu FB . 2012. Red meat consumption and mortality: results from 2 prospective cohort studies. Arch Intern Med 172:555–563. doi:10.1001/archinternmed.2011.2287 22412075 PMC3712342

[13] Abete I , Romaguera D , Vieira AR , Lopez de Munain A , Norat T . 2014. Association between total, processed, red and white meat consumption and all-cause, CVD and IHD mortality: a meta-analysis of cohort studies. Br J Nutr 112:762–775. doi:10.1017/S000711451400124X 24932617

[14] Micha R , Michas G , Lajous M , Mozaffarian D . 2013. Processing of meats and cardiovascular risk: time to focus on preservatives. BMC Med 11:136. doi:10.1186/1741-7015-11-136 23701737 PMC3680013

[15] Chan DSM , Lau R , Aune D , Vieira R , Greenwood DC , Kampman E , Norat T . 2011. Red and processed meat and colorectal cancer incidence: meta-analysis of prospective studies. PLoS One 6:e20456. doi:10.1371/journal.pone.0020456 21674008 PMC3108955

[16] Farvid MS , Sidahmed E , Spence ND , Mante Angua K , Rosner BA , Barnett JB . 2021. Consumption of red meat and processed meat and cancer incidence: a systematic review and meta-analysis of prospective studies. Eur J Epidemiol 36:937–951. doi:10.1007/s10654-021-00741-9 34455534

[17] Wang M , Wang Z , Lee Y , Lai HTM , de Oliveira Otto MC , Lemaitre RN , Fretts A , Sotoodehnia N , Budoff M , DiDonato JA , McKnight B , Tang WHW , Psaty BM , Siscovick DS , Hazen SL , Mozaffarian D . 2022. Dietary meat, trimethylamine N-oxide-related metabolites, and incident cardiovascular disease among older adults: the cardiovascular health study. ATVB 42. doi:10.1161/ATVBAHA.121.316533 PMC9420768 35912635

[18] Zhu Y , Jameson E , Crosatti M , Schäfer H , Rajakumar K , Bugg TDH , Chen Y . 2014. Carnitine metabolism to trimethylamine by an unusual rieske-type oxygenase from human microbiota. Proc Natl Acad Sci U S A 111:4268–4273. doi:10.1073/pnas.1316569111 24591617 PMC3964110

[19] Craciun S , Marks JA , Balskus EP . 2014. Characterization of choline trimethylamine-lyase expands the chemistry of glycyl radical enzymes. ACS Chem Biol 9:1408–1413. doi:10.1021/cb500113p 24854437

[20] Nishihara K , Kanemori M , Kitagawa M , Yanagi H , Yura T . 1998. Chaperone coexpression plasmids: differential and synergistic roles of DnaK-DnaJ-GrpE and GroEL-GroES in assisting folding of an allergen of Japanese cedar pollen, Cryj2, in Escherichia coli . Appl Environ Microbiol 64:1694–1699. doi:10.1128/AEM.64.5.1694-1699.1998 9572938 PMC106217

[21] Nishihara K , Kanemori M , Yanagi H , Yura T . 2000. Overexpression of trigger factor prevents aggregation of recombinant proteins in Escherichia coli. Appl Environ Microbiol 66:884–889. doi:10.1128/AEM.66.3.884-889.2000 10698746 PMC91917

