# Supplementary material for: Assembling the anaerobic gamma-butyrobetaine to TMA metabolic pathway in Escherichia fergusonii and confirming its role in TMA production from dietary L-carnitine in murine models
Source: mBio. 2023 Sep 22;14(5):e00937-23. doi: 10.1128/mbio.00937-23 (PMC10653785; doi:10.1128/mbio.00937-23)
Supplement: Supplemental Information — Supplemental methods and Fig. S1–S6. [file mbio.00937-23-s0001.pdf]

Supplementary Information

Assembling the anaerobic gamma-butyrobetaine to TMA metabolic pathway in *Escherichia fergusonii* and confirming its role in TMA production from dietary *L*-carnitine in murine models.

Mohammed Dwidar[1,2,3*], Jennifer A. Buffa[1,2], Zeneng Wang[1,2], Akeem Santos[1,2], Aaron N. Tittle[1,2], Xiaoming Fu[1,2], Adeline M. Hajjar [1,2,3], Joseph A. DiDonato[1,2,3], and Stanley L. Hazen[1,2,3,4].

[1] Department of Cardiovascular & Metabolic Sciences, Lerner Research Institute, Cleveland Clinic, Cleveland, OH

[2] Center for Microbiome & Human Health, Cleveland Clinic, Cleveland, OH

[3] Cleveland Clinic Lerner College of Medicine, Case Western Reserve University, Cleveland, OH

[4] Department of Cardiovascular Medicine, Heart, Vascular and Thoracic Institute, Cleveland Clinic, Cleveland, OH

[*] Corresponding author. Mohammed Dwidar, Email: dwidarm@ccf.org;
Lerner Research Institute, Cleveland Clinic, 9500 Euclid Avenue, Cleveland, Ohio 44195

Table of contents:

Supplementary methods

Supplementary figures S1-S6

Supplementary references

Supplementary Methods:

Bacterial strains and culturing conditions

Escherichia fergusonii ATCC 35469 was used in all experiments. Wild-type and recombinant *E. fergusonii* strains were kept as frozen glycerol stocks and cultured on LB (Luria-Bertani) media for all *in-vitro* experiments. When needed, Kanamycin and/or ampicillin were added to the cultures at concentrations of 50, and 100 µg/ml, respectively, and d_9-ɣBB, d_3-*L*-carnitine, and d_6-choline were added at concentrations of 100~200 µM.

Chromosomal knock-in of *gbu* gene cluster in *E. fergusonii*

Synthetic codon-optimized genes corresponding to *gbuA, B, C,* and *E* genes from *Emergencia timonensis* SN18 were cloned in a suicide plasmid harboring a kanamycin-resistant gene cassette, R6K origin, and *sacB* gene. The two homology recombination arms of the plasmid were designed to insert the operon coding for the *gbu* genes in-between the native *caiE*, and the *caiF* genes so that the *gbu* operon was placed immediately downstream of the *caiE* gene, and in front of a predicated native downstream terminator. This location was chosen to allow for potential enhancement of the *gbu* operon expression by the upstream *cai* operon promotor. The suicide plasmid was propagated in *E. coli* S17-1λpir, then transferred to *E. fergusonii* through conjugation. The successful transconjugants were obtained through plating the conjugation mixture on LB plates supplemented with kanamycin and ampicillin, as *E. fergusonii* is naturally resistant to ampicillin. The purified transconjugants were then plated on LSW (1) agar plates supplemented with 10% sucrose to select for the successful knock-in mutants, which were then purified and verified through PCR, Sanger sequencing, and their ability to grow

in the presence of ampicillin but not kanamycin. The composition of the LSW agar plates (per liter) was 10 g tryptone, 5 g yeast extract, 5 mL glycerol, 0.4 g NaCl, and 20 g agar.

Mass spectrometry analysis:

Hydrochloric acid at a final concentration of 60 mM was added to the bacterial culture samples immediately after collection to prevent the loss of TMA through evaporation. [$^{13}C_3$, ^{15}N]-TMA was used as internal standard for quantitation of TMA and its isotopologues d_3-TMA, d_6-TMA and d_9-TMA, and the samples were processed by hexane/butanol extraction under alkaline pH followed by acidification and transfer to aqueous phase as described previously (2). For measuring d_3-*L*-carnitine, d_9-ɣBB, d_3-ɣBB, and d_6-choline, bacterial culture supernatants were mixed with the same volume of ɣBB, d_4-choline and d_9-*L*-carnitine with a concentration of 20 µM each, and filtered through a 3KDa cut-off membrane filters (Amicon®, UFC5003BK). The filtrate was injected onto LC/MS-MS for quantitation, and ɣBB was used as internal standard for d_3-ɣBB and d_9-ɣBB, while d_9-carnitine and d_4-choline were used as internal standards for d_3-carnitine and d_6-choline, respectively.

Serum samples for TMA, TMAO and other TMA-related metabolites and their isotopologues following d_3-carnitine, d_6-choline and d_9-butyrobetaine challenge were processed by adding four volumes of methanol containing 5 µM d_4-choline, 2 µM [$^{13}C_3$]-TMAO and 2 µM [$^{13}C_3$,^{15}N]-TMA. [$^{13}C_3$]-TMAO and [$^{13}C_3$, ^{15}N]-TMA were used as internal standards for their corresponding isotopologues, while d_4-choline was used for all other metabolites and their corresponding isotopologues (3).

1 µl of the prepared samples were injected onto LC column through Shimadzu autosampler (SIL-HTc) and metabolites were resolved on silica column (Luna, 00F-4274-B0, Phenomenx) with LC gradients generated from binary pumps (Shimadzu LC-20AD) connected to two solvents, A: 0.1% propionic acid in water; B: 0.1% acetic acid in methanol; starting from 0% B in the first 2 minutes, followed by linear increasing to 15% over 3 minutes, then to 100% over 5 minute, holding at 100% B for 3 minutes, then finally returning back to 0% B to equilibrate column for 3 minutes. The LC flow rate was 0.2 ml/min. The LC elutes were analyzed on API 5000 Mass spectrometer (AbSciex) with an electrospray ion source. Standards and internal standards were monitored in positive multiple reaction monitoring mode with parent to daughter transitions determined by standards, and MS parameters were optimized by individual standards. Standard curves were generated from serial dilutions of standards undergoing the same procedures as real samples.

Germ-free mouse colonization

All experiments involving mice were performed using protocols approved by the Cleveland Clinic Animal Care and Use Committee. Germfree female C57BL/6 mice were bred at the Cleveland Clinic gnotobiotic animal facilities in a controlled environment in plastic flexible film gnotobiotic isolators (Class Biologically Clean, Ltd., Madison, WI) under a strict 14h light/10h dark cycle, and received sterilized non-acidified water and food (LabDiet, Catalogue # 5010 - Laboratory Autoclavable Rodent Diet) *ad libitum*. This diet contains choline at 1800 ppm per manufacturer data sheet. The chow was not supplemented with any additional dietary precursors for TMA. For experiments, mice

were housed in Allentown Sentry Sealed Positive Pressure cages (Allentown, NJ) and handled according to methods shown previously (4).

Frozen glycerol stocks of *E. fergusonii* recombinant strains were administered as ~0.2 mL oral gavage into 15-20 week old mice inside a biological safety cabinet. Mice were maintained on a sterilized diet and their drinking water was supplemented with kanamycin sulfate (50 µg/mL) and either *L*-carnitine (1.3% w/v) or ɣBB (1.6% w/v). Kanamycin was added to ensure the stability of the pGro plasmid in the recombinant *E. fergusonii* strains. After 1 week, serum and urine samples were collected to measure TMAO, *L*-carnitine and ɣBB levels, and then tracer studies were performed. The investigator was not blinded to treatment groups to avoid cross contamination between groups.

***In vivo* tracer studies**

After colonization with the recombinant *E. fergusonii* strains, mice received a 200 µl oral gavage containing 112 mM each of d_3-*L*-carnitine, d_9-γbb, and d_6-choline in a biological safety cabinet. Blood was collected from the saphenous vein at the indicated time. Whole blood was centrifuged to isolate serum, which was stored at -80°C until analyzed. Serum levels of endogenous and stable isotope-labeled *L*-carnitine, γBB, choline, TMA, and TMAO were determined using LC-MS/MS. Laboratory personnel performing MS analyses were blinded to sample group allocation during analysis.

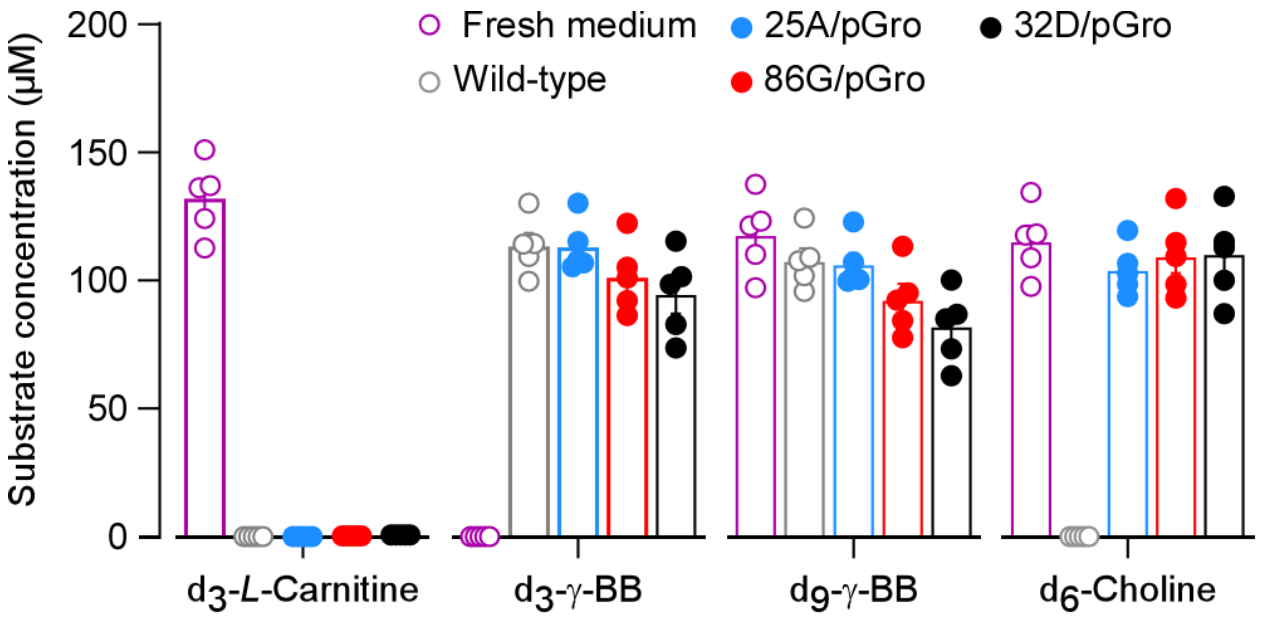

Figure S1. Substrate consumption levels for figure 2E. Wild-type and mutant *E. fergusonii* strains were cultured under anaerobic conditions at 37 ºC in the presence of d_3-carnitine, d_9- γBB, and d_6-choline. Samples were taken after 48 h for LC-MS/MS analyses (n=5). Bars represent mean +/- SE.

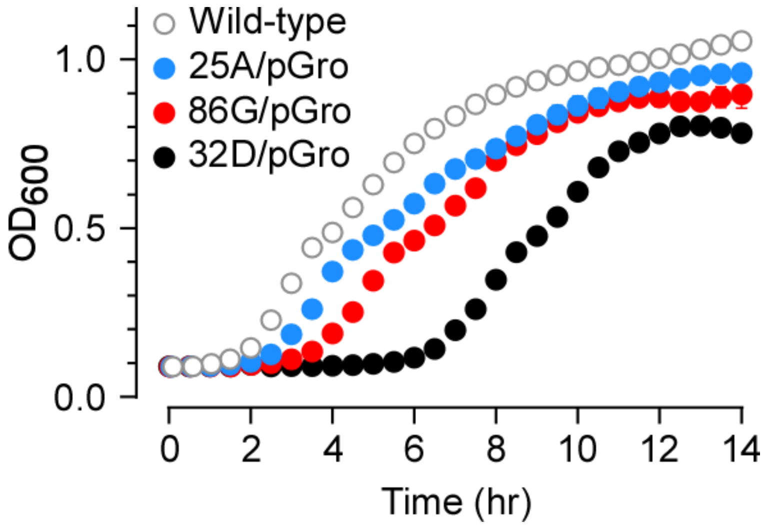

Figure S2. Growth curve of wild-type *E. fergusonii* and mutants when grown in LB media. All strains were diluted from overnight cultures (1:500) in fresh LB media, and aliquots from each were analyzed in 96-well plate (200 µl per well). OD_{600} was measured over time in microplate reader. The LB media was supplemented with kanamycin at 50 µg/ml for 25A/pGro, 86G/pGro, and 32D/pGro to ensure plasmid stability. Points represent mean +/- SE. **(**n=5)

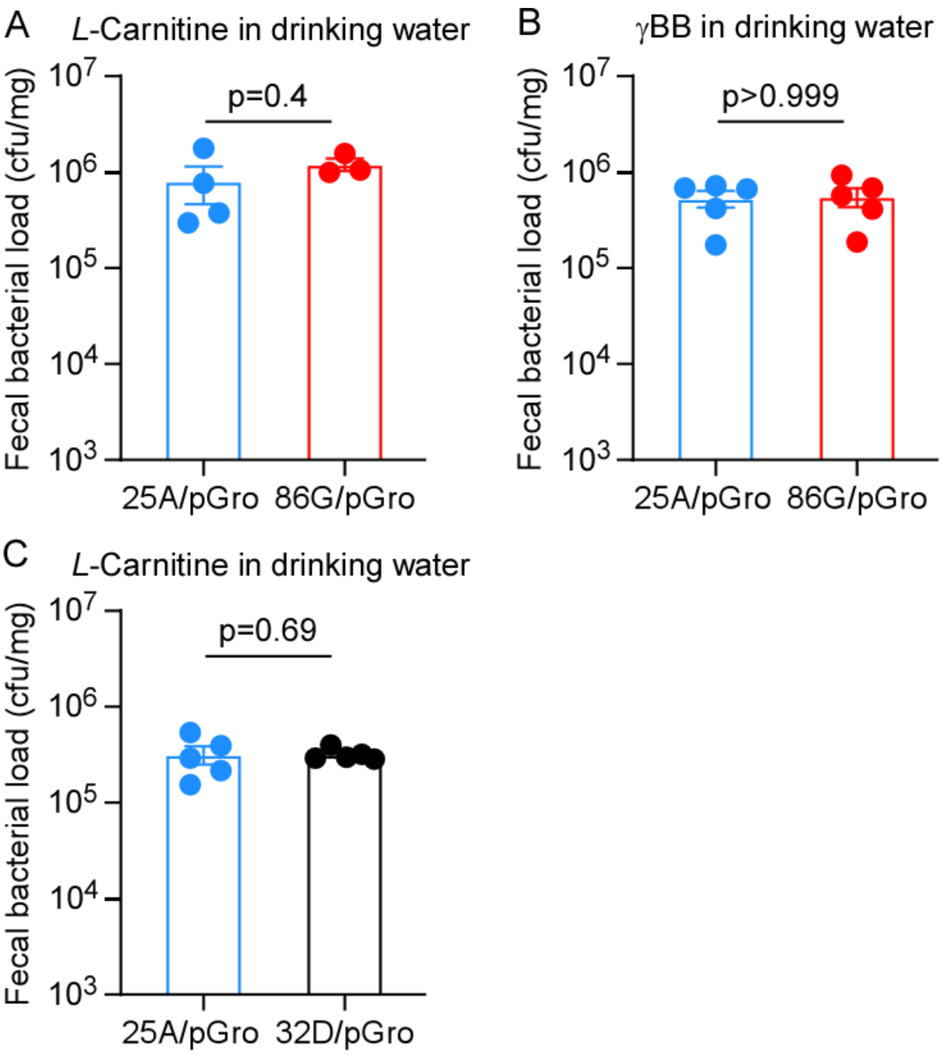

Figure S3. The gain-of-function mutants *E. fergusonii* 86G/pGro and 32D/pGro show colonization comparable to the control 25A/pGro strain. (A, B) Germ-free mice were randomized and inoculated with either the gain-of-function mutant 86G/pGro or the control strain 25A/pGro, and provided either *L*-carnitine or γbb in the drinking water. (C) In an independent experiment, germ-free mice were inoculated with 32D/pGro or the control strain 25A/pGro and given *L*-carnitine. Fecal samples were taken at day 7 after colonization, weighed, diluted, and plated on LB agar plates supplemented with both ampicillin and kanamycin for counting. Bars represent mean +/- SE. Reported are p-values from Mann Whitney test. (n=3~5)

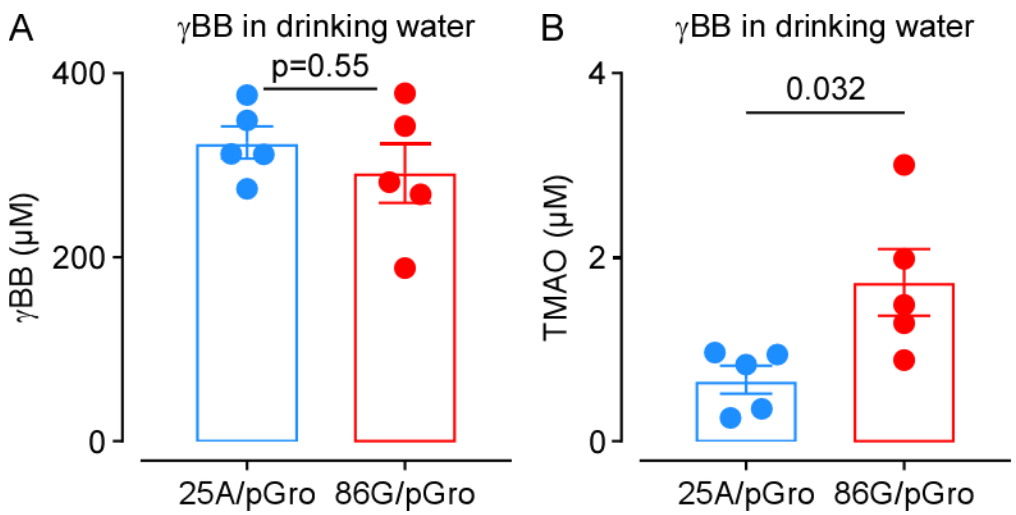

Figure S4. Serum levels of ɣBB and TMAO from mice colonized with either *E. fergusonii* 86G/pGro or *E. fergusonii* 25A/pGro after ɣBB supplementation. Mice were kept on drinking water supplemented with 1.6% w/v ɣBB for 7 days after colonization. Reported are p-values from Mann Whitney test. Bars represent mean +/- SE. (n= 5)

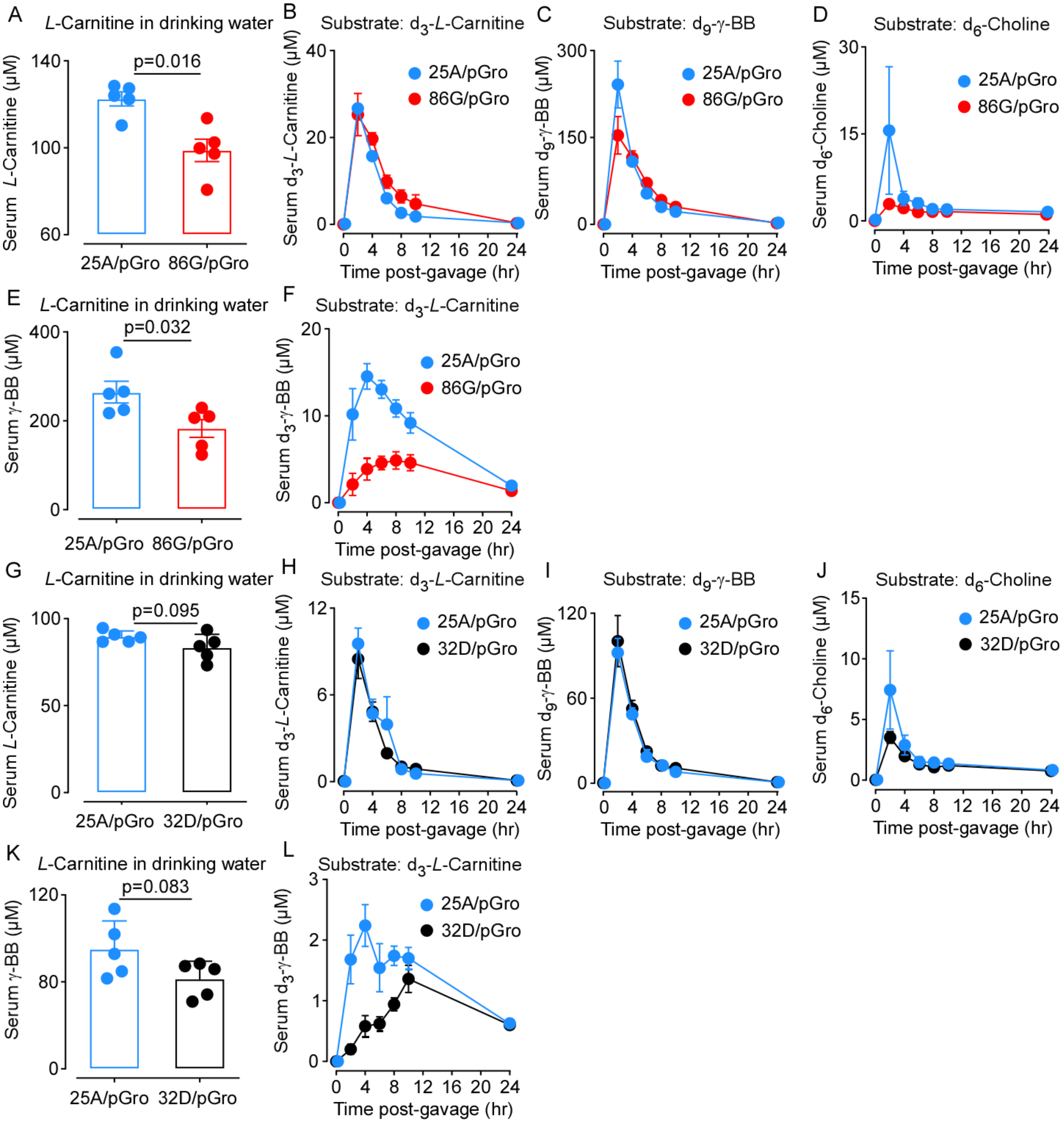

Figure S5. Serum substrate levels of mice shown in Figure 2.

(A-F) The gnotobiotic mice harboring 86G/pGro or 25A/pGro (control) were fed *L*-carnitine in the drinking water (at 1.3%) for 1 week, after which serum levels of *L*-carnitine, and ɣBB were measured using LC-MS/MS. Mice from both groups were then gavaged with a single bolus of d_6-choline + d_3-*L*-carnitine + d_9-ɣBB, and their plasma was analyzed at

different time points after gavage. (G-L) The same experiment was repeated using gnotobiotic mice harboring 32D/pGro strain for the test group and 25A/pGro strain for the control. n=5 for each group in both experiments. Reported are p-values from Mann Whitney test. Bars represent mean +/- SE.

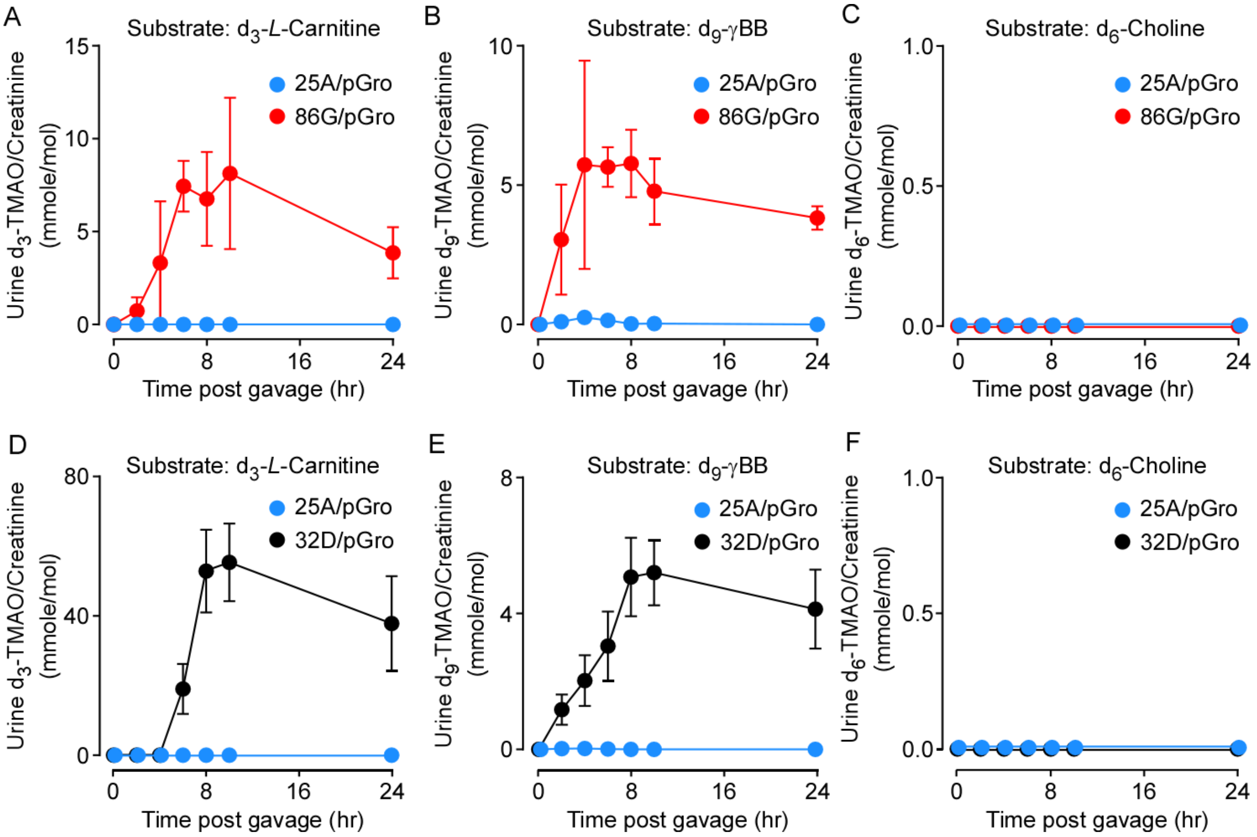

Figure S6. Colonization of germ-free mice with recombinant *E. fergusonii* expressing *gbuABCE* cassette and *groES/groEL*-like genes increases urine TMAO levels upon exposure to d_3-*L*-carnitine or d_9-ɣBB.

(A-C) The gnotobiotic mice harboring 86G/pGro or 25A/pGro (control) were given *L*-carnitine in the drinking water (at 1.3%) for 1 week. Mice from both groups were then gavaged with a single bolus of d_6-choline + d_3-*L*-carnitine + d_9-ɣBB, and urine TMAO/Creatinine levels were assessed at different time points after gavage. (D-F) The same experiment was repeated using gnotobiotic mice harboring 32D/pGro strain for the test group and 25A/pGro strain for the control. n=3-5 per group. Bars represent mean +/- SE.

Supplementary References

1. Howery KE, Rather PN. 2019. Allelic Exchange Mutagenesis in Proteus mirabilis. Methods Mol Biol 2021:77-84.
2. Jia X, Osborn LJ, Wang Z. 2020. Simultaneous Measurement of Urinary Trimethylamine (TMA) and Trimethylamine N-Oxide (TMAO) by Liquid Chromatography-Mass Spectrometry. Molecules 25, 1862
3. Wang Z, Bergeron N, Levison BS, Li XS, Chiu S, Jia X, Koeth RA, Li L, Wu Y, Tang WHW, Krauss RM, Hazen SL. 2019. Impact of chronic dietary red meat, white meat, or non-meat protein on trimethylamine N-oxide metabolism and renal excretion in healthy men and women. Eur Heart J 40:583-594.
4. Paik J, Pershutkina O, Meeker S, Yi JJ, Dowling S, Hsu C, Hajjar AM, Maggio-Price L, Beck DA. 2015. Potential for using a hermetically-sealed, positive-pressured isocage system for studies involving germ-free mice outside a flexible-film isolator. Gut Microbes 6:255-65.
